# Advancing developmental biology in Central Europe: report from the V4SDB meeting 2025

**DOI:** 10.1242/bio.062360

**Published:** 2025-12-11

**Authors:** Petra Kompaníková, Kriti Attri, Dávid Czimer, Anastasiia Hubiernatorova, Ewelina Trela-Kobędza, David Zimčík

**Affiliations:** ^1^ Department of Experimental Biology, Faculty of Science, Masaryk University, Kamenice 5, 625 00 Brno, Czech Republic; ^2^ Institute of Molecular Genetics of the Czech Academy of Sciences, Prague 4, Czech Republic; ^3^ Department of Genetics, ELTE Eötvös Loránd University, 1117 Budapest, Hungary; ^4^ Department of Embryology, Institute of Developmental Biology and Biomedical Sciences, Faculty of Biology, University of Warsaw, 02-096, Warsaw, Poland; ^5^ Institute of Animal Physiology and Genetics, Czech Academy of Sciences, 602 00 Brno, Czech Republic

**Keywords:** Visegrád Group Society for Developmental Biology, V4SDB, Developmental biology, Early development, Organogenesis, Evo-devo, Stem cells

## Abstract

Hosted in the scenic surroundings of the High Tatras, the V4SDB Meeting 2025 represented the fourth biennial conference of the Visegrád Group Society for Developmental Biology. The meeting brought together over 170 researchers covering a multidisciplinary range of topics, from early development and organogenesis to non-traditional model systems and evo-devo, as well as stem cells and plasticity. With its numerous high-level talks and rich poster sessions, complemented by social and outreach activities, the meeting provided an excellent platform for networking, knowledge exchange, and initiating new collaborations. Moreover, the organizers arranged the Young Developmental Biologists Workshop, focused on data analysis, soft-skills training and community strengthening, which further enhanced early-career researchers' participation and support at the meeting. Altogether, the event underscored the V4SDB's commitment to nurturing the developmental biology community within the region.

## Teaser

**Summary:** The V4SDB 2025 Meeting embodies a vibrant Central European developmental biology community fostering innovation, advancing new tools and ideas, and building the next generation of young scientists.

## Introduction

Developmental biology is an intriguing fundamental discipline that seeks to understand how a single cell gives rise to a complex, fully functional organism. The field addresses essential biological questions, elucidates mechanisms and provides concepts operating at the levels of whole organisms, organs, and tissues – the scales at which many human diseases originate and manifest. Thus, developmental biology is indispensable for understanding congenital malformations or infertility and is equally vital for advancing tissue engineering, regeneration and even cancer research (Prokop, 2017; Wallingford, 2019).

Given its broad scope and impact, supporting the community of developmental biologists and fostering new generations of scientists remain essential. The Visegrád Society of Developmental Biology (V4SDB) was founded in 2018 with this mission – to create a unifying association for developmental biologists, strengthen the field's presence across the Visegrád region and enhance collaboration and visibility, filling a long-standing gap in Central Europe.

Since then, V4SDB has grown into a vibrant platform serving the community through mini symposia, student schools and, most prominently, its biennial meeting, the society's flagship event that brings together researchers from the region and beyond.

Following three successful meetings in Czechia, Hungary, and Poland (Trela and Walewska, 2023), the V4SDB 2025 Meeting was held on 5-7 September 2025 in Stará Lesná, Slovakia, completing the full hosting cycle across all four Visegrád countries. Organized by Zuzana Sumbalová Koledová (Institute of Molecular Genetics of the Czech Academy of Sciences, Czechia), Dušan Fabian and Alexandra Špirková (both Centre of Biosciences of the Slovak Academy of Sciences, Institute of Animal Physiology, Slovakia), Marcela Buchtová (Institute of Animal Physiology and Genetics of the Czech Academy of Sciences, Czechia and Masaryk University, Czechia) and Petra Kompaníková (Masaryk University, Czechia), the meeting featured internationally recognized invited speakers alongside numerous excellent contributed talks and posters.

**Fig. 1.**
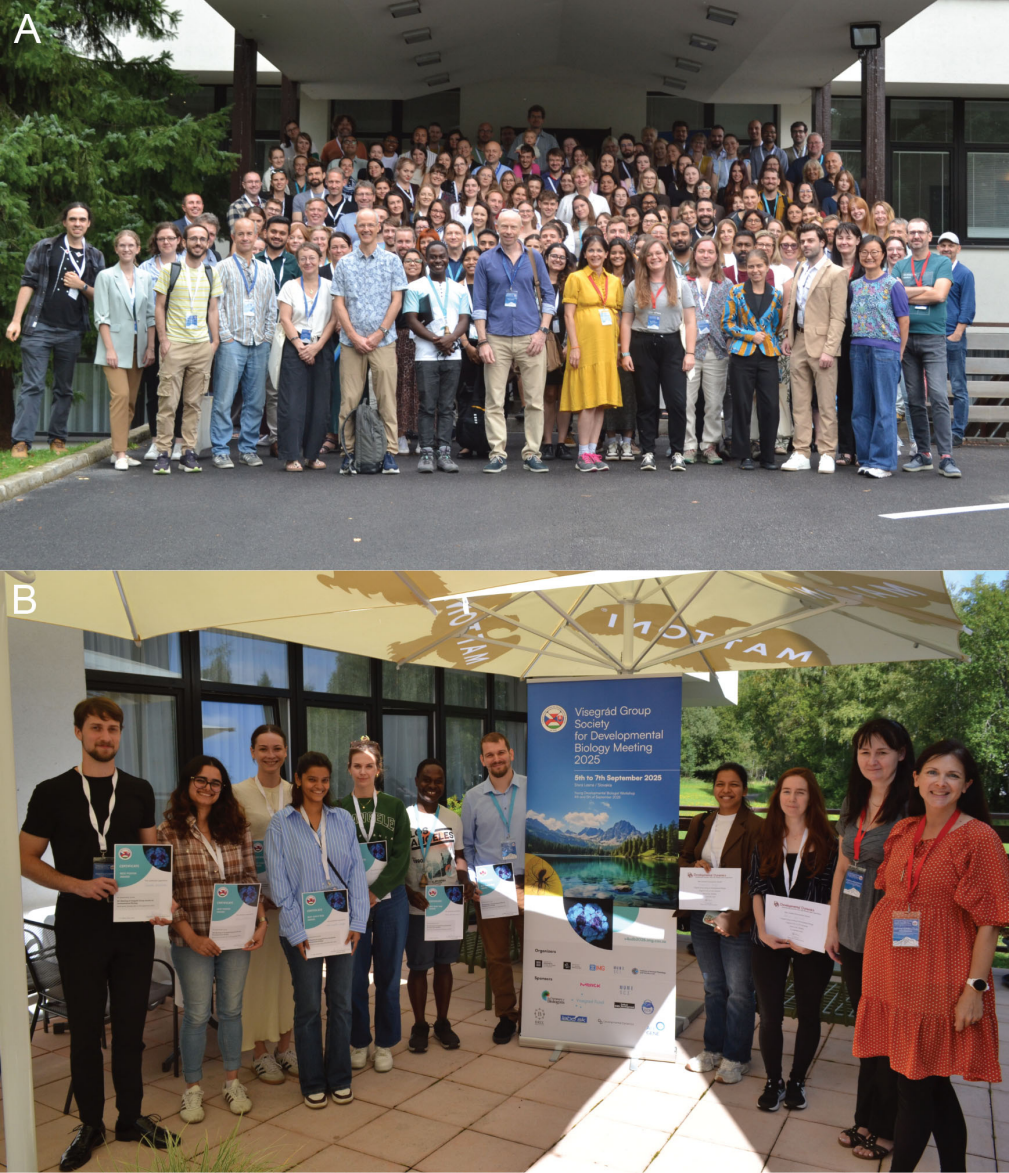
**Moments from the V4SDB Meeting 2025.** (A) Group photo of the V4SDB meeting participants. (B) Awardees of the best oral, chalk talk, and poster presentations. From left to right: Tomáš Suchánek (Charles University, Czechia), Kriti Attri (Institute of Molecular Genetics of the Czech Academy of Sciences, Czechia), Małgorzata Wojtaszek (Medical University of Gdańsk, Poland), Afifah Azimuddin Chogle (Institute of Molecular Genetics of the Czech Academy of Sciences and Charles University, Czechia), Marcelina Osyra (Poznań University of Life Sciences, Poland), Allan Akandwanaho (Institute of Molecular Genetics of the Czech Academy of Sciences, Czechia), Ádám Soós (Semmelweis University, Hungary), Himanshi Singh (Charles University, Czech Republic), Denisa Belisová (Masaryk University, Czechia), Marcela Buchtová, V4SDB President, and Zuzana Sumbalová Koledová, Chair of the Conference.

Over 170 researchers (Fig. 1A) from different countries, career stages, and areas of expertise took part, reflecting the society's inclusive and collaborative spirit. The program featured 27 talks and 116 posters, encompassing all major areas of developmental biology, from early development and organogenesis to evolutionary and stem cell biology, with emerging themes and methodological advances summarized in the sections below.

## Zooming in on the next generation

A defining feature of all V4SDB events is the strong presence and active involvement of early-career researchers. The V4SDB has consistently emphasized fostering the next generation of developmental biologists, providing them with visibility, training opportunities, and a supportive international platform.

In this spirit, a day before the main conference, the Young Developmental Biologists Workshop was held to promote collaboration, skill development, and collective learning among early-career researchers. PhD students and postdoctoral fellows presented their projects through short chalk talks, encouraging lively discussion and peer feedback. The workshop also included a series of hands-on sessions designed to address the diverse backgrounds and interests of participants. These sessions covered a range of essential skills, from effective scientific abstract writing to advanced data analysis, including time-lapse imaging, single-cell transcriptomics, proteomics, micro-CT analysis or even an AlphaFold prediction session. Additionally, a roundtable discussion on the role of artificial intelligence in research was organized by Máté Varga (ELTE Eötvös Loránd University, Hungary), where students and invited panelists – Igor Adameyko (Medical University of Vienna, Austria and Karolinska Institutet, Sweden) and Gage Crump (University of Southern California, USA) – explored how to effectively navigate and integrate these emerging tools into modern scientific practice.

At the main meeting, early-career researchers were further engaged through several short oral presentations and flash talks, providing an opportunity to showcase their work to a broader audience. Outstanding presentations, posters and chalk talks were recognized with awards, reflecting the society's ongoing commitment to celebrating and empowering emerging scientists (Fig. 1B). The meeting also provided the opportunity to establish the Young Researcher Committee of V4SDB, tasked to further promote the society's efforts to reach out the early-career researchers.

## Shaping organs: how complex form and function emerge?

Moving on to the meeting, one of its central themes was the need for an interdisciplinary approach that unites experimental biology with computational modeling to elucidate how complex organs and tissues acquire their precise shape, form, and function.

James Sharpe (EMBL Barcelona, Spain) presented his group's data-driven computational models for understanding limb development (Uzkudun et al., 2015) and introduced his latest initiative, LimbNet (Matyjaszkiewicz and Sharpe, 2024 preprint) – an online platform that enables researchers to build, simulate, and share spatiotemporal models of limb morphogenesis within a common framework. He emphasized that such integrative modeling approaches are essential for capturing the complexity of organ formation, linking processes across multiple biological scales, from gene regulatory networks and cellular behaviors to tissue-level morphogenetic dynamics, and for tracing the causal relationships that connect these levels.

The complexity of tissue development across these scales was further underscored by several talks addressing the interplay between molecular signaling and morphogenesis. Jakub Harnoš (Masaryk University, Czechia) presented data for potential functional interaction between key Wnt pathway components and the Notch intracellular domain during embryonic epidermis development. Ondřej Machoň (Institute of Experimental Medicine, Czech Academy of Sciences, Czechia) demonstrated how deletion of *Wnt5a* in neural crest cells affects polarized cellular components leading to craniofacial malformations, similar to human developmental defects such as Robinow syndrome (Burianova et al., 2025 preprint).

Another multidisciplinary perspective on organ formation was provided by Anne Grapin-Botton (Max Planck Institute of Molecular Cell Biology and Genetics, Germany). Combining live imaging, computational modeling and genetic perturbations, her group investigates how, during pancreatic development, cellular and tissue architecture affects cells' fate choices and the dynamics of these processes. Besides her recent work focusing on the roles of lumen formation and hydrostatic pressure in shaping the tissue (Lu et al., 2025), Anne also highlighted a novel organoid system that both mimics architectural complexity and simplifies experimental manipulation (Tanida et al., 2025).

The necessity of appropriate tools and model systems to advance our understanding of tissue formation and its potential malformations crystallized as another theme of the meeting. Peter Fabian (Masaryk University, Czechia) presented a new zebrafish model for studying alkaptonuria and metabolic role of dermal fibroblasts in phenylalanine and tyrosine degradation (Fabian et al., 2022), linking local metabolism to the tissue pathology observed in the given disease. Another methodological innovation, which now enables precise gene editing in chickens, was introduced by Jiří Hejnar (Institute of Molecular Genetics, Czech Academy of Sciences, Czechia). By combining primordial germ cell transplantation with CRISPR-based genome editing, his work has elegantly overcome longstanding barriers in avian reverse genetics (Koslová et al., 2020). Jan Křivánek (Masaryk University, Czechia) introduced BEE-ST, a novel method for monitoring hard tissue growth across species, emphasizing its broad applicability in developmental biology, bone and tooth repair, tissue engineering, and disease modeling (Lopez et al., 2023). Complementing the hard tissue topic, Maria Hovoraková (Charles University, Czechia) provided new insight into bone-forming processes by tracing the progeny of *Sonic hedgehog*-expressing cells during mouse jaw embryonic development, contributing to the understanding of bone regeneration and potential therapeutic strategies.

## Abundance of information – what can we learn from early development?

Spanning oocyte biology, lineage specification or even developmental dormancy, the Early Development session illustrated both the robustness and adaptability of the earliest stages of life. Beyond just advancing our understanding of early developmental control, the talks also revealed mechanisms that could one day underpin advances in infertility treatments and reproductive medicine.

Lenka Gahurová (University of South Bohemia, Czechia) offered an evolutionary view of reproductive longevity, showing that African mole-rats maintain fertility through unique oocyte and ovarian-niche features that preserve genome stability and resist aging, bridging toward mechanisms regulating early embryonic development.

Focusing on oocytes from the perspective of maturation, Martin Anger (Institute of Animal Physiology and Genetics, Czech Academy of Sciences, Czechia) examined the molecular regulation of meiosis, suggesting that the Anaphase-Promoting Complex, though restrained by the spindle assembly checkpoint, is active during meiosis I and targets important cell cycle regulators (Horakova et al., 2024). Anna Piliszek (Institute of Genetics and Animal Biotechnology, Polish Academy of Sciences, Poland) addressed the later stage of development, trophectoderm specification in a rabbit embryo. She demonstrated that ROCK activity is crucial for distinguishing trophectoderm from the inner cell mass (Filimonow et al., 2025).

Invited speaker of the session, Ivan Bedzhov (Max Planck Institute for Molecular Biomedicine, Germany), expanded the session by delving into diapause – a mechanism by which certain species cease or slow early embryo growth and reactivate it under advantageous circumstances. Using mouse models and single-cell RNA sequencing, his group revealed that three lineages of embryo exhibit transcriptional dynamics during entry, maintenance, and exit from the dormancy. Additionally, both embryonic and extraembryonic tissues preserve their cell identity and stem cell properties by halting their differentiation programs, with integrin-Yap/Taz signaling governing their survival and reactivation (Chen et al., 2024).

Finally, Allan Akandwanaho (Institute of Molecular Genetics, Czech Academy of Sciences, Czechia) discussed the establishment of the left-right asymmetric body plan through torque-generated rotatory movements in *C. elegans*. He discussed the role of RhoA signaling in active torque generation and the molecular activity of CYK-1/Formin molecules in chiral rotatory movements.

## Organ evolution and the emergence of novelty

Across the Non-traditional Models and Evo-Devo sessions, the spotlight turned to the fascinating processes underlying vertebrate diversity, innovation, and adaptation during evolution, and to how these phenomena can be uncovered through unconventional model systems, theoretical frameworks, quantitative approaches and the latest technologies.

Michel C. Milinkovitch (University of Geneva, Switzerland) showcased to the audience the power of mathematical–phenomenological models in explaining the diversity of vertebrate skin colors and appendage forms. In detail, he focused on Turing's reaction–diffusion systems and discrete cellular automata, which capture complex biological pattern formation without requiring exhaustive molecular detail. Integrating growth dynamics and skin geometry, his group explained color pattern transitions in ocellated lizards (Fofonjka and Milinkovitch, 2021). Turning to crocodilians, he contrasted chemical Turing-type mechanisms driving body-scale formation with purely mechanical instabilities underlying head-scale patterning, captured by simple elastic models (Santos-Durán et al., 2025).

Máté Varga provided another striking example of evolutionary innovation, the labyrinth organ. This specialized structure enables aerial respiration and reshapes both physiology and behavior in the paradise fish (Szabó et al., 2024). His group is now applying modern molecular and imaging approaches to unravel the processes underlying its formation.

Another theme of the Non-traditional Models and Evo-Devo sessions focused on the origins and diversification of vertebrate craniofacial structures, highlighting how comparative approaches and various model systems are reshaping our understanding of vertebrate evolution.

Gage Crump illustrated how the mammalian external ear evolved through the repurposing of ancestral gill developmental programs. Using single-cell multiomic profiling and cross-species enhancer assays, his team demonstrated that the external ear and fish gills share conserved gene regulatory networks. These findings strongly support the view that evolutionary innovation in craniofacial structures often arises from modifications of existing developmental programs rather than the invention of entirely new regulatory mechanisms (Thiruppathy et al., 2025).

Iryna Kozmiková (Institute of Molecular Genetics, Czech Academy of Sciences, Czechia) discussed the evolutionary origins of vertebrate head structures. Her work demonstrated that amphioxus embryos possess prechordal plate-like and neural crest-like cell populations regulated by conserved Nodal, Hedgehog, and Wnt pathways, suggesting that core elements of vertebrate head development were already present in the common chordate ancestor (Markos et al., 2024).

Building on this evolutionary theme, Ann Huysseune (Charles University, Czechia and Ghent University, Belgium) and Agata Horáčková (Charles University, Czechia) used basal fish models – Senegal bichir, sterlet sturgeon, and tropical gar – to explore the evolution of craniofacial and endocrine structures, respectively. Huysseune's team described complex ectoderm–endoderm interactions during pre-oral gut development, which is important for understanding the development and evolution of the vertebrate oropharynx derivatives and their disorders, while Horáčková showed that these fishes retain developmental and functional traits linking the vertebrate adenohypophysis to ancestral exocrine pharyngeal glands, echoing characteristics of the proto-pituitary in amphioxus. These results illuminate a key evolutionary transition from exocrine to endocrine function in the vertebrate head, providing a deeper understanding of how a central regulatory gland emerged.

Abigail Tucker (King's College London, UK and Charles University, Czechia) traced the evolution of mammalian suckling mechanisms by studying soft palate development in monotremes and therians. Comparative analyses identified conserved skeletal elements, such as the hamulus, and revealed that muscle activity is essential for proper palate formation, shedding light on the anatomical innovations that enabled efficient suckling in mammals.

## The flexibility of cells

Later sessions highlighted how stem cellular plasticity, responding to developmental or environmental cues, empowers tissues to form, renew and repair themselves, and how the same flexibility, when misdirected, can contribute to disease.

Using single-cell RNA sequencing combined with pioneering barcoding-based clonal lineage tracing in mouse embryos and machine learning, Igor Adameyko revealed a continuous gradient of fate biases, where spatial position and clonal composition are tightly interconnected. This work innovatively challenges the traditional tree-like model of cell differentiation, highlighting instead a dynamic landscape of multipotency shaped by both intrinsic transcriptional programs and extrinsic signaling cues (Erickson et al., 2025 preprint).

Complementing this developmental perspective, Krzysztof Kobielak and Anna Puławska-Czub (both University of Warsaw, Poland) highlighted the conserved roles of BMP signaling in epithelial stem cell regulation. Kobielak discussed the interplay between Bmp and Id2 in maintaining stem cell quiescence and activation within the hair follicle niche, while Puławska-Czub demonstrated that modulating BMP signaling, either through its activation or inhibition, leads to enhanced or impaired regeneration of the digit tip following amputation. Further insights into regeneration came from Radek Šindelka (Institute of Biotechnology of the Czech Academy of Sciences, Czechia), who presented his recent work on a newly identified cell population, regeneration initiating cells, which are responsible for extracellular matrix post-amputation organization during the early stages of *Xenopus laevis* tail regeneration (Sindelka et al., 2024).

Bringing this session into the context of disease, Han Li (Institut Pasteur, France) introduced her work on mammary gland involution and the role of cellular senescence as a key process in remodeling of this tissue. Interestingly, while mammary involution mimics wound healing, it also creates a transient pro-tumorigenic microenvironment strongly implicated in postpartum breast cancer. She further showed that involution-induced senescence enhances the plasticity of tumor-initiating cells, promoting invasion and metastasis (von Joest et al., 2022; Chiche et al., 2024 preprint).

Irina Poverennaya (Medical University of Vienna, Austria) expanded the discussion by introducing the role of rRNA modifications in biasing cell fate decisions of cranial neural crest cells highlighting also the pathological significance of rRNA-modifying and ribosomal assembly genes in patients with neuroblastoma. Lastly, Ádám Soós (Semmelweis University, Hungary) demonstrated that growth factor supplementation enhances avian enteric neurosphere generation, offering potential therapeutic applications for disorders such as Hirschsprung disease.

## Conclusions

The V4SDB Meeting 2025 demonstrated the remarkable creativity of developmental biologists who combine classical experimentation and model systems with cutting-edge technologies, computational modeling and unconventional organisms to reveal how organisms are formed and how the endless forms of structures have emerged through evolution. Equally important was the strong participation of enthusiastic early-career researchers, offering confidence that developmental biology in the region will continue to thrive. This will no doubt be reaffirmed at the next meeting, to be held in Prague in 2027, marking the beginning of a new geographic cycle for V4SDB meetings.
